# A robust circular RNA-based prognostic signature for postoperative recurrence in stage II/III colon cancer

**DOI:** 10.3934/genet.2019.4.67

**Published:** 2019-09-30

**Authors:** Ji Ruan

**Affiliations:** State Key Laboratory of Oncology in South China, Collaborative Innovation Center for Cancer Medicine, Sun Yat-sen University Cancer Center, Guangzhou 510060, China

Colon cancer is one of the leading causes of mortality and morbidity worldwide [1], with a dramatic rise in incidence and mortality rates over the past 3 decades, both worldwide and in China [2]. Surgery is the principal therapy for early-stage colon cancer because it offers the only method for complete tumor removal, thereby chance for cure [3]. However, there is still proportions of colon cancer patients that develop recurrence even after radical resection [4]. Unfortunately, long term control of the recurrent colon cancer has been a difficult dilemma to tackle and the clinical outcomes of these patients are poor [5],[6]. Adjuvant chemotherapy in colon cancer is able to complement curative surgery to reduce the risk of recurrence and death from relapsed or metastatic disease [7],[8]. However, patient willingness to be adjuvantly treated has been limited due to its associated financial burden, treatment toxicity and debatable prolongation of survival that need to be carefully balanced against the associated-toxicity. Therefore, the identification of robust biomarkers to assess the risk of postoperative recurrence in colon cancer has been a longing need.

Circular RNAs (circRNAs), a recently discovered type of noncoding RNA, have a special circular structure formed by 3′- and 5′-ends linking covalently [9]. Because they do not have 5′ or 3′ ends, they are resistant to exonuclease-mediated degradation [10]. Therefore, circRNAs are characterized by their high stability, expression level, and evolutionary conservation. Recent literatures [11]–[13] have confirmed circRNAs as playing an important role in cancer initiation and progression. Additionally, they are differentially expressed in cancer tissues and circulation of cancer patients. Due to these characteristics, circRNAs have gained recognition as a promising novel biomarker for cancer [14]. However, till now little is known about circRNAs and their relationship with colon cancer. In a recent study published in *EMBO Molecular Medicine*, entitled “A circRNA signature predicts postoperative recurrence in stage II/III colon cancer”, Ju et al. [15] identified and validated a circRNA-based signature that could improve postoperative prognostic stratification of patients with stage II/III colon cancer.

In that study, 437 colon cancer circRNAs were examined in tumoral and adjacent normal tissues. The authors found that 103 circRNAs were differentially expressed in recurrent colon cancer patients as compared to non-recurrent counterparts. Among them, 100 up-regulated circRNAs were validated in patients with stage II/III colon cancer to test whether they could be used as prognostic biomarkers. Four circRNAs, including hsa_circ_0122319, hsa_circ_0087391, hsa_circ_0079480, and hsa_circ_0008039, showed strong predictive values for disease-free survival (DFS) in the validation cohort. The following circRNA-based prognostic model was thereby generated:

$$cirScore=0.\text{46}\times {\text{Exp}}_{hsa\_circ\_0122319}+(-0.\text{386}\times {\text{Exp}}_{hsa\_circ\_0083791})+0.\text{293}\times {\text{Exp}}_{hsa\_circ\_0079480}+0.\text{439}\times {\text{Exp}}_{hsa\_circ\_0008039}$$

After adjusting for baseline clinicopathological factors, the cirScore was found suitable for serving as predictive biomarkers of postoperative recurrence in stage II/III colon cancer. Patients in the high-risk group (cirScore ≥ −0.323) had poorer DFS (hazard ratio [HR], 2.89, 95% confidence interval [CI], 1.37–6.09, *P* < 0.001) and overall survival (OS; HR, 4.22, 95% CI, 1.61–11.03, *P* < 0.001) than those in the low-risk group (cirScore < −0.323). Based on this circRNA-based prognostic model, the authors further established a nomogram which exhibited good performance for estimating the 3- or 5-year DFS and OS.

To further explore the biological functions of hsa_circ_0122319, hsa_circ_0087391, and hsa_circ_0079480 (the abundance of hsa_circ_0008039 was found to be low in colon cancer cell line was therefore not further explored), the authors performed a series of functional *in vitro* and *in vivo* experiments. They found that suppression of these three circRNAs in colon cancer cells could significantly alter *in vitro* migration capacity. Additionally, the knockdown of hsa_circ_0079480 in colon cancer cells could both inhibit *in vivo* lung and liver metastasis.

The strengths of this study include the well-designed identification approaches, mature clinical data with clinical outcome and long-term follow-up, and well-characterized tissue samples for colon cancer. Limitations included that the prognostic model was only applicable for the patients with stage II/III colon cancer. In summary, this study identified a 4-circRNA-based expression signature as highly predictive for cancer recurrence in stage II/III colon cancer, with the potential of identification of high-risk early-stage colon cancer individuals who would benefit most from adjuvant chemotherapy.
